# Natural history of Morquio A patient with tracheal obstruction from birth to death

**DOI:** 10.1016/j.ymgmr.2017.11.005

**Published:** 2017-12-22

**Authors:** Caitlin Doherty, Lauren W. Averill, Mary Theroux, William G. Mackenzie, Christian Pizarro, Robert W. Mason, Shunji Tomatsu

**Affiliations:** a Nemours/Alfred I. duPont Hospital for Children, Wilmington, DE, USA; b University of Delaware, Newark, DE, USA; c Department of Pediatrics, Gifu University, Gifu, Japan; d Department of Pediatrics, Thomas Jefferson University, Philadelphia, PA, USA

**Keywords:** Natural history, Morquio A syndrome, Tracheal obstruction, Autopsy, Keratan sulfate

## Abstract

Morquio A syndrome (mucopolysaccharidosis IVA, MPS IVA) is a lysosomal storage disease caused by a deficiency of *N*-acetylgalactosamine-6-sulfate sulfatase, resulting in systemic accumulation of the partially degraded glycosaminoglycans (GAGs), keratan sulfate and chondroitin-6-sulfate. The accumulation of these GAGs leads to distinguishing features as skeletal dysplasia with disproportionate dwarfism, short neck, kyphoscoliosis, pectus carinatum, tracheal obstruction, coxa valga, genu valgum, and joint laxity. In the absence of autopsied cases and systemic analysis of multiple tissues, the pathological mechanism of the characteristic skeletal dysplasia associated with the disease largely remains a question. Here we report an autopsied case of a 23-year-old male with MPS IVA, who developed characteristic skeletal abnormalities by 4 months of age and died of severe tracheal obstruction and hypoventilation originating from respiratory muscle weakness from neurological cord deficit due to cord myelopathy at the age of 23. We analyzed postmortem tissues pathohistologically, including the thyroid, lung, lung bronchus, trachea, heart, aorta, liver, spleen, kidney, testes, humerus, knee cartilage, and knee ligament.

Examination of the tissues demonstrated systemic storage materials in multiple tissues, as well as severely ballooned and vacuolated chondrocytes in the trachea, humerus, knee cartilage, and lung bronchus.

This autopsied case with MPS IVA addresses the importance of tracheal obstruction for morbidity and mortality of the disease, and the pathological findings contribute to a further understanding of the pathogenesis of MPS IVA and the development of novel therapies.

## Highlights

• Severe tracheal obstruction and respiratory failure are the leading causes of morbidity and mortality in MPS IVA patients.
• The majority of chondrocytes in the examined locations were enlarged and vacuolated.
• Tracheal obstruction is confirmed clinically, pathologically, and radiographically.
• Risk factors and surgical intervention of tracheal obstruction should be considered to save the lives of MPS IVA patients.
• MPS IVA should be evaluated in a multifaceted approach.

## 1 Introduction

Morquio A syndrome (mucopolysaccharidosis IVA, MPS IVA) is a lysosomal storage disease (LSD) caused by a deficiency of *N*-acetylgalactosamine-6-sulfate sulfatase (GALNS), which is required for the catabolism of glycosaminoglycans (GAGs): keratan sulfate (KS) and chondroitin-6-sulfate (C6S) [1], [2], [3], [4], [5], [6], [7]. As a result, partially degraded GAGs accumulate in bone, ligaments, and cartilage, as well as the extracellular matrix (ECM) of these tissues, impeding endochondral ossification and chondrogenesis [2], [3], [8]. In looking at the bone pathology of MPS IVA, endochondral ossification is primarily distorted at articular and growth cartilage [2], [8], [9]. MPS IVA ranges from mild to severe, with systemic bone dysplasia often increasing the severity of the disease [8], [9]. MPS IVA is characterized by extensive clinical manifestations including skeletal dysplasia with prominent forehead, dental abnormalities, short neck, short trunk dwarfism, cervical spinal cord compression and atlantoaxial instability, tracheal obstruction, kyphoscoliosis, pectus carinatum, pulmonary complications, laxity of joints, coxa valga, genu valgum, and elevated blood and urine KS [1], [2], [3], [8], [9]. In addition, radiographic findings display failure of ossification, cervical spinal stenosis with dysplasia of odontoid process, platyspondyly of vertebral bodies, flaring of the rib cage, anterior beaking of the lumbar bodies, tilted ulna, epiphyseal dysplasia of joints, flaring iliaca, and flattened femoral head. While most patients appear normal at birth, major skeletal abnormalities often develop within a few years of age [1]. Individuals with MPS IVA, particularly with the severe form, often do not survive past their twenties, as the most attributed causes of mortality and morbidity are spinal cord compression, instability of the C1-C2 joint, and airway compromise including tracheal obstruction [1], [2], [3], [8], [9].

Therefore, most patients require several surgeries to alleviate severe orthopedic complications, such as cervical spinal decompression and fusion, limb osteotomy, hemiepiphysiodesis, and hip reconstruction/replacement; nonetheless, they often become wheelchair-dependent by their second decade [1], [2], [3], [8], [9].

Tracheostomy has often been required for tracheal obstruction, and due to a difficult airway, patients remain high risk for anesthesia, making intubation and extubation challenging or impossible [3], [10]. Difficult airways in MPS IVA patients arise from the imbalance of growth between the trachea and vessels vs. cervicothoracic spine and ribs, short neck, large tongue and mandible, hypertrophic adenoid and tonsils, and the angle of manubrium as a result of GAG deposits in chondrocytes and their ECM [2], [17], [18]. A study examining tracheal obstruction in 28 MPS IVA patients with the severe phenotype revealed that 67.9% of patients showed tracheal narrowing, and that tracheal narrowing worsened with age (all 8 patients over the age of 15 had > 50% tracheal narrowing [3], [7]. Eight out of the 28 patients presented with severe (> 75%) tracheal narrowing. This tracheal narrowing was found to be most attributed to compression by the tortuous brachiocephalic artery and was evident as early as at the age of 2 years old. Performing a tracheostomy can prove difficult in MPS IVA patients due to a tortuous and redundant trachea, short neck, and inability to hyperextend the neck. Airway obstruction due to vascular compression and a narrowed thoracic inlet requires an alternative method. Pizarro et al. describe a novel surgical procedure for tracheal obstruction without the need for a tracheostomy, which should improve difficult airway and narrowed trachea [11].

As MPS IVA remains a systemic skeletal disorder without a cure, there have been few histological and molecular evaluations of cartilage and bone pathology in MPS IVA patients, particularly due to the lack of autopsied cases [2]. While two autopsied cases were presented in the 1970s, only brain pathology was reported, and neither case was diagnosed enzymatically [2], [12], [13]. A fetal case with MPS IVA described in 1992 showed that electron microscopy (EM) depicted that placental villi and resting chondrocytes have multiple vacuoles, with the accumulation of storage materials starting in the fetus. Biopsied cartilage of MPS IVA patients indicates a high expression of collagen type I and a low expression of collagen type II, with the speculation that this type of collagen expression seen could lead to laxity of joints [2], [14]. Cartilage ECM of MPS IVA patients is also affected, impacting the phenotypic properties of chondrocytes, and causing the development of cartilage that is more prone to degradation [2], [15]. This provides an explanation for the early occurrence of osteoarthritis. It was also recently proposed that the inadequate regression of cartilage canals and impaired resorption and restitution of pannus tissue could be the cause of early pathogenesis in MPS IVA [2], [16]. In 2013, Yasuda et al. reported pathologic and morphologic findings of tissues from an autopsied case with MPS IVA 8 years post-failed HSCT, which was the first incidence in which the pathology of multiple tissues was described systemically.

Currently, we are unable to fully explain the pathogenic mechanism of the characteristic skeletal dysplasia associated with MPS IVA as well as the widespread involvement of other tissues [2]. In order for the development of therapies that could benefit patients with MPS IVA, it is imperative that we gain comprehensive knowledge regarding the pathogenesis of the disease through the examination of affected tissues.

In this article, we have described the pathologic observations of tissues from an autopsied case with severe tracheal obstruction and respiratory muscle weakness arising from neurological cord deficit due to cord myelopathy to further understand the pathogenesis of MPS IVA.

## 2 Materials and methods

### 2.1 Tissues analyzed

A 23-year-old male with MPS IVA was autopsied at the University of Tennessee. Informed consent for the autopsy was obtained, and tissue preparations for the pathological analyses were conducted at Alfred I. duPont Hospital for Children. We examined postmortem tissues including the heart, aorta, liver, trachea, thyroid, lung, lung bronchus, spleen, kidney, testes, humerus, knee ligament, and knee cartilage by light microscopy (LM).

### 2.2 Light microscopy (LM)

Bone and soft tissue samples were fixed for 24–36 h in 10% neutral buffered formalin. Bone samples were then decalcified in Regular Cal Immuno™ (BBC Biochemicals, Mount Vernon, WA), washed in running water, and examined for end-point decalcification. Tissues were auto-processed through graded ethanol, cleared in Safe-Clear™ (Thermo-Fisher, Kalamazoo, MI), and then embedded in paraffin. 5 μm sections were cut, floated onto poly-lysine coated slides, and heat-immobilized at 60° C for 1 h. Prior to staining, slides were cooled to room temperature. Sections were deparaffinized, hydrated with distilled water, and stained with colloidal iron/ van Gieson, hematoxylin and eosin (H&E), Alcian blue/periodic acid-Schiff (PAS), toluidine blue (TB), and Safranin O (Saf O). Using a standard H&E protocol, H&E stains were conducted on a Sakura DRS-601 automated stainer. Colloidal iron stains were performed manually through the use of a modified Mowery's technique [2]. The Alcian Blue/PAS staining method is as follows: tissues were placed in an Alcian blue solution of pH 2.5 for 30 min. Excess stain was removed from the sides and then placed in a periodic acid solution for 10 min. The sections were rinsed in running tap water for 5 min, placed in Schiff's reagent for 10 min, and washed for another 10 min in lukewarm water. For Safranin O, tissues were stained with Safranin O and fast green. All of the slides were dehydrated in graded ethanol, cleared in Histo-Clear™ (National Diagnostics, Atlanta, GA) and cover-slipped in permount. Colloidal iron stains acidic mucopolysaccharides a dark blue colour, while van Gieson stains collagen pink. H&E staining demonstrates the morphology of the cell, with hematoxylin staining nuclei in blue and eosin staining the cytoplasm in pink. Alcian blue staining turns acidic mucopolysaccharides blue, while PAS staining turns sugars and neutral mucopolysaccharides red. Safranin O stains nuclei black, the cytoplasm gray/green, and cartilage and acidic proteoglycans red.

For evaluation of lysosomal storage by light microscopy, toluidine blue-stained 0.5-μm thick sections were examined. The formula of fixative for toluidine blue (TB) is as follows; 0.1 M cacodylic acid formaldehyde (final ca.1.5%) and glutaraldehyde (final 1%) (pH 7.2–7.4 adjusted with HCl). Following infiltration with EmBed resin, blocks were polymerized at 80 °C, and thin sections were cut with Leica EM UC7 ultramicrotome.

## 3 Results

### 3.1 Case report (Fig. 1, Fig. 2)

This Caucasian patient was born without complications at 38-weeks gestation, with a birth weight and length of 2.72 kg and 43.2 cm, respectively. While he began sitting at 5 to 6 months of age, crawling at 7 to 8 months and walking at 13 months, thoracolumbar kyphosis and mild dextroconvex scoliosis were detected at 4 months of age. By 2 years old, the patient had exhibited further anomalies including lordosis and short stature, which led to an enzymatically confirmed diagnosis of MPS IVA at the age of 3 years and 2 months old at the University of Tennessee, Medical Center. At the time of his diagnosis, the patient had shown worsening kyphosis and scoliosis as well as vision and hearing difficulties, eventually requiring glasses and bilateral hearing aids. Prominent forehead, hearing loss, corneal clouding, dental problems, large tongue, short neck, chest deformities, laxity of joints, and decreased stamina developed with age. The patient was found to have severe cervical cord compression at the C-1 level at 4 years old, resulting in posterior decompression and occipito-C1-C2 fusion surgery, which was successful (Fig. 4). It was also noted that the patient displayed osteopenic bones with unusually broad and shortened tibia and metatarsals, thin bone cortices, and tibiotalar slant in his ankles. By 5 years of age, the patient stopped growing with his maximal height reaching 86.4 cm, and by 6 years of age, he displayed platyspondyly. By the age of 12, the patient had fair strength and endurance although he presented with severe malalignment of his knees with a greater valgus deformity in the left knee, causing his left pelvis to be lower than the right. During this time, the patient was able to walk independently on even surfaces; however, he became short of breath upon exertion. Due to his joint deformities and small size, the patient was unable to maneuver stairs.

**Fig. 1:**
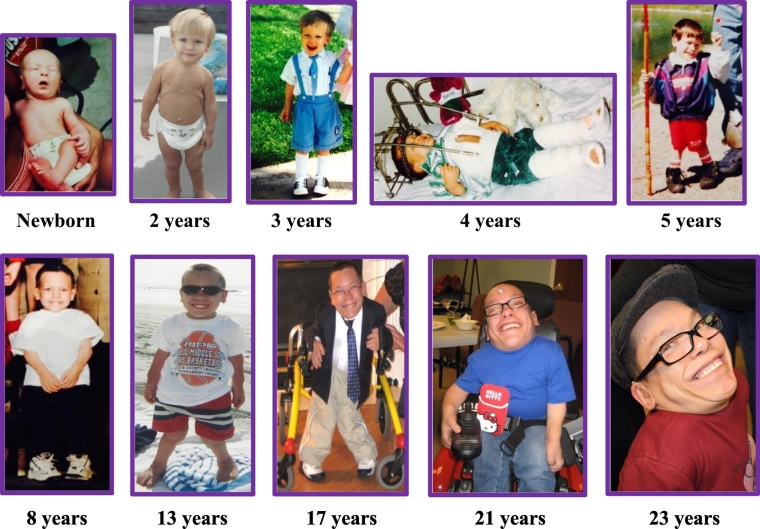
The clinical course of an autopsied Morquio A syndrome patient. The catalogued case depicts the characteristic skeletal deformities present in patients with Morquio syndrome with hyperlaxity of joints, pectus carinatum, and genu valgum (knock-knee). The patient displayed noticeable short stature with pectus carinatum at age 2, genu valgum at age 5, stopped growing around the age of 5 (height: 86.4 cm; body weight: 19 kg at 18 years old) as well as hyperlaxity of joints. His posture (the look up at the sky position) seen at ages 21 and 23 is typical of Morquio patients to ease breathing.

**Fig. 2:**
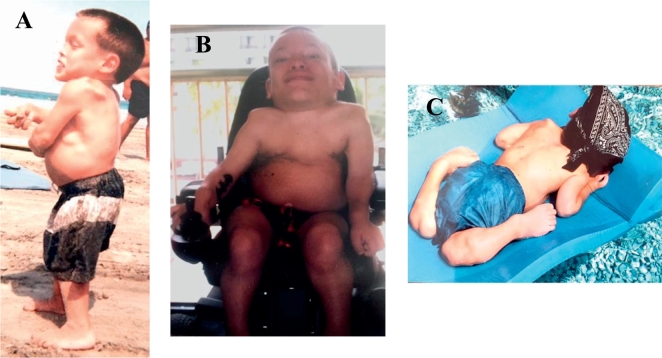
Clinical manifestations associated with Morquio A syndrome. (A) A side view of the patient at age 10 shows a prominent forehead, pectus carinatum, hyperlaxity of joints, kyphoscoliosis, genu valgum, and short trunk stature. (B) At age 19, the patient exhibits the look up to the sky position, a short neck, pectus carinatum, and hyperlaxity of joints. (C) The patient at age 21 displays hyperlaxity of joints.

At age 16, the patient began to experience a severe decrease in bilateral lower extremity strength and sensation, which then inhibited ambulation and caused minor bowel and bladder incontinence. MRI of the cervical spine showed spinal stenosis as well as marked tracheal narrowing with triangular configuration at the thoracic inlet (Fig. 4).

A CT scan of the thoracolumbar spine at the age of 17 revealed lumbar stenosis at the T12-L1 level, prompting thoracolumbar decompressive laminectomy surgery at the T11-L2 level and posterior spinal fusion surgery at the T10-L4 level (Fig. 5). The patient regained the ability to ambulate by postoperative day 5; however, by 6-months post-decompression and fusion surgery, the patient was only able to walk up to a distance of 61 m with a walker, and exhibited poor lower body strength and endurance. At 10-months post-decompression and fusion surgery, the patient had lost control over his bowel and bladder, as well as the ability to ambulate, becoming permanently wheelchair-bound. During this time, the patient experienced difficulty breathing and began holding his head in extension as described by the “look up to the sky position.” Similar to the MRI from a year earlier, CT showed focal triangular-shaped narrowing of his trachea at the thoracic inlet, spanning approximately 3 cm craniocaudal (Fig. 5). The cross-sectional area of the trachea just below the vocal cords, at the thoracic inlet, and 2 cm above the carina measured 109 mm^2^, 20 mm^2^, and 66 mm^2^, respectively. This corresponds to an 81.7% reduction in the tracheal cross-sectional area at the thoracic inlet compared to the cervical trachea, and a 66.7% reduction compared to the intrathoracic trachea. Mild tortuosity of the trachea in the coronal plane was also present.

By the age of 17.5 years, the patient's symptoms became progressive in nature, beginning with a numbness and decrease in sensation in his left hand that advanced to the left buttocks region, and the patient's mobility became limited to minimal motion of his fingers at the age of 18.5 years. From this point on, the patient remained bedridden, experiencing a further onset of neurological, musculoskeletal, and respiratory manifestations, including sleep apnea and the look up to the sky position. The patient's condition worsened until his death at the age of 23, during which he was short of breath with diminishing respiration efficiency. The progressive manner of the patient's disease in combination with his clinical manifestations was indicative that he had the severe form of the disease. It is noteworthy that the patient with MPS IVA died of respiratory failure with severe tracheal obstruction and hypoventilation originating from his respiratory muscle weakness as a result of cervical spinal cord myelopathy, which contributes to the findings seen upon examination of the patient's tissues. Severe degree of cervical cord myelopathy and resultant respiratory weakness was evident from the neurological symptoms that the patient exhibited.

### 3.2 Autopsy report

**Fig. 3:**
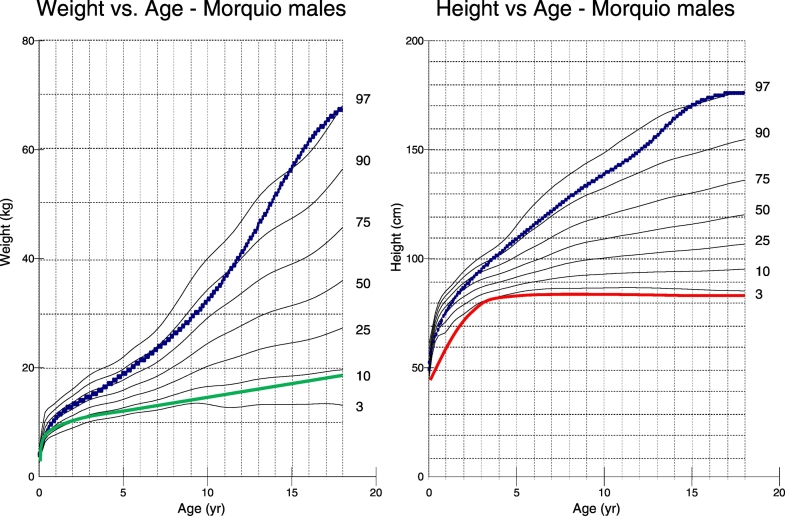
Stature and Weight-for-age percentile for autopsied Morquio patient as compared to other Morquio patients [19]. (A) The patient stopped growing around 5 years of age, reaching a max height of 86.4 cm. (B) At 18 years of age, the patient reached a body weight of 19 kg. The patient was exceptionally shorter and weighed less as compared to other MPS IVA patients, suggestive of the fact that the patient had the severe phenotype of the disease. The dotted line shows the 50th percentile for normal males.

The height of the patient was 86.4 cm, which is − 13 SD as compared to the healthy male height from the Center for Disease Control and Prevention (CDC) growth chart. His weight was 19 kg, which is − 7 SD as compared to healthy male weight. As compared to MPS IVA male height and weight, the patient was slightly above the 3rd percentile and in the 10th percentile, respectively (Fig. 3). The patient's body mass index (BMI) was 25.5 kg/m^2^. The weights and relative sizes of the patient's organs were normal.

**Fig. 4:**
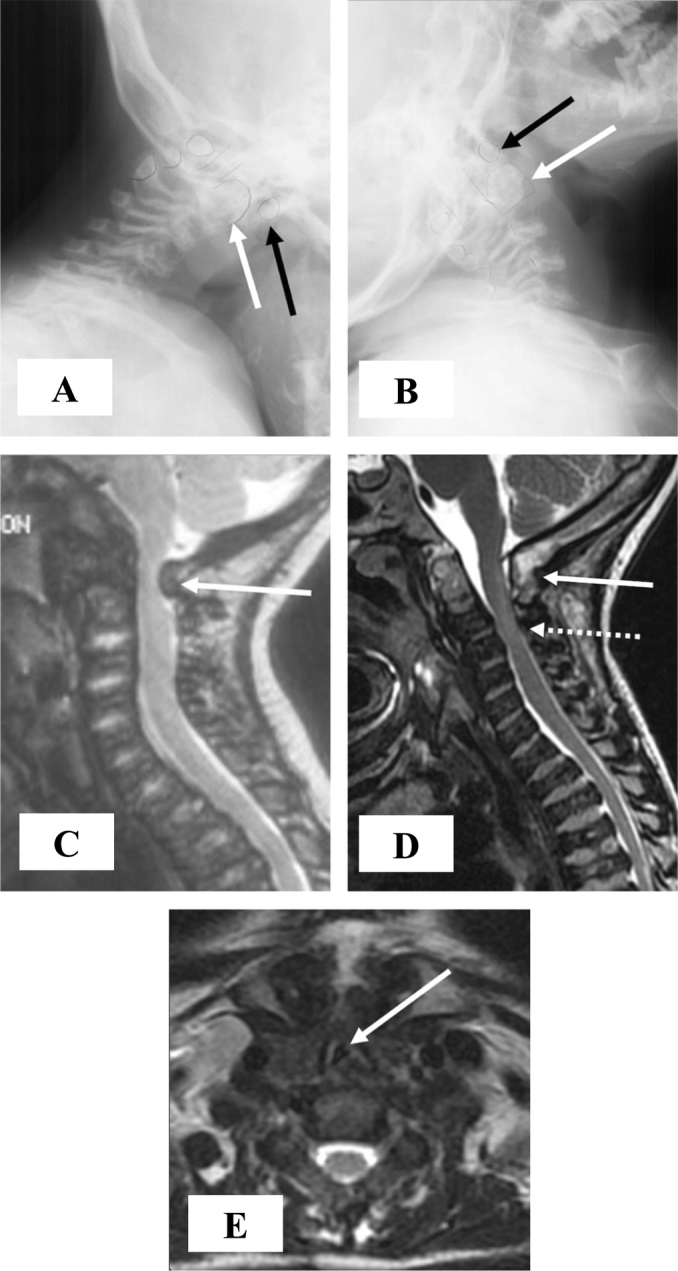
Pre- and postoperative imaging of the cervical spine. Preoperative cervical spine x-rays at age 3 years performed in flexion (A) and extension (B) show atlantoaxial instability, with bony outlines and spinal canal highlighted in pencil. The anterior arch of C1 (black arrows) rides up of the hypoplastic dens (white arrows) in extension, and moves anteriorly in flexion causing decreased space available for the spinal cord. (C) A preoperative sagittal T2-weighted MR image of the cervical spine at age 4 years shows spinal cord impingement by the posterior arch of C1 (arrow). Occipital to C2 posterior decompression and fusion was performed shortly afterwards. (D) A subsequent sagittal T2-weighted MR image of the cervical spine at age 16 years shows mature occipital to C2 bony fusion (arrow) with diffuse spinal stenosis at lower levels (dashed arrow) but no spinal cord gliosis. (E) An axial T2-weighted MR image from the same study shows marked tracheal narrowing with triangular configuration (arrow) at the thoracic inlet.

**Fig. 5:**
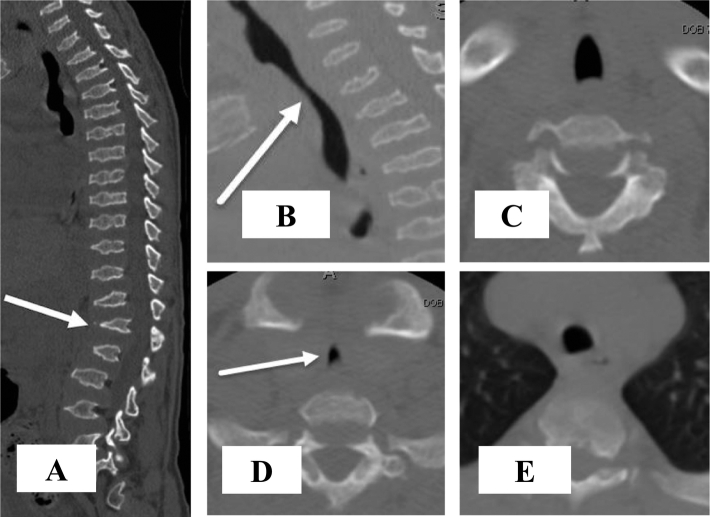
CT images of the spine, with attention to the vertebrae and trachea. A midline sagittal CT image of the spine (A) at 17 years of age shows typical features of MPS IVA leading to severe spinal stenosis, including: platyspondyly, endplate irregularity, anterior beaking of vertebral bodies, and mild retrolisthesis of L1 (arrow). T10-L4 spinal decompression and fusion was subsequently performed. Coned down sagittal image (B) from the same study with attention to the trachea shows narrowing of the trachea at the thoracic inlet (arrow), spanning approximately 3 cm craniocaudal. Coned down axial images just below the vocal cords (C), at the thoracic inlet (D), and 2 cm above the carina (E) show focal triangular shaped narrowing of the trachea (arrow) at the thoracic inlet. Tracheal cross-sectional area measures 109 mm^2^, 20 mm^2^, and 66 mm^2^ at these respective levels. There is mild tortuosity of the trachea in the coronal plane as well, not possible to show in a single image due to the oblique course and lack of 3D data set for multiplanar reconstruction. Narrowing of the thoracic inlet is due to bony changes of MPS IVA, including pectus carinatum and bulbous clavicular heads, as well as soft tissue crowding. Detailed vascular anatomy as a contributing factor cannot be delineated on this non-contrast CT of the spine.

**Fig. 6:**
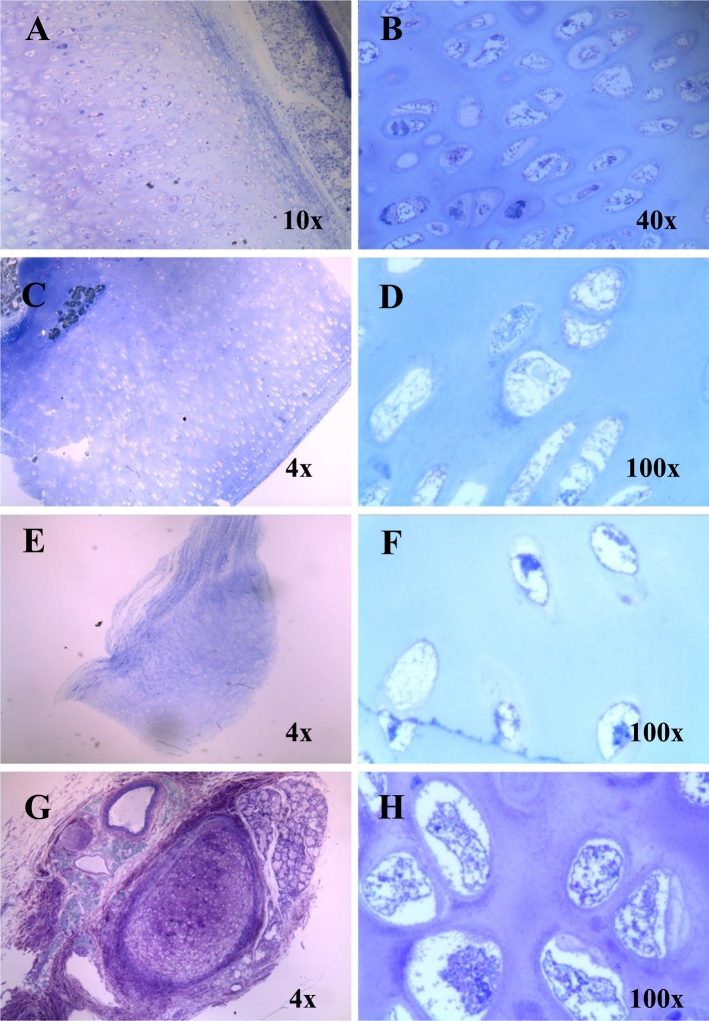
Chondrocytes in the trachea, humerus, knee cartilage, and lung bronchus stained with toluidine blue (light microscopy). (A) and (B) Trachea shows enlarged and vacuolated chondrocytes. (C) and (D) The right humerus displays ballooned and vacuolated chondrocytes. (E) and (F) Knee cartilage shows vacuolated chondrocytes. (G) and (H) Bronchus in the left lung indicates enlarged vacuolated chondrocytes.

**Fig. 7:**
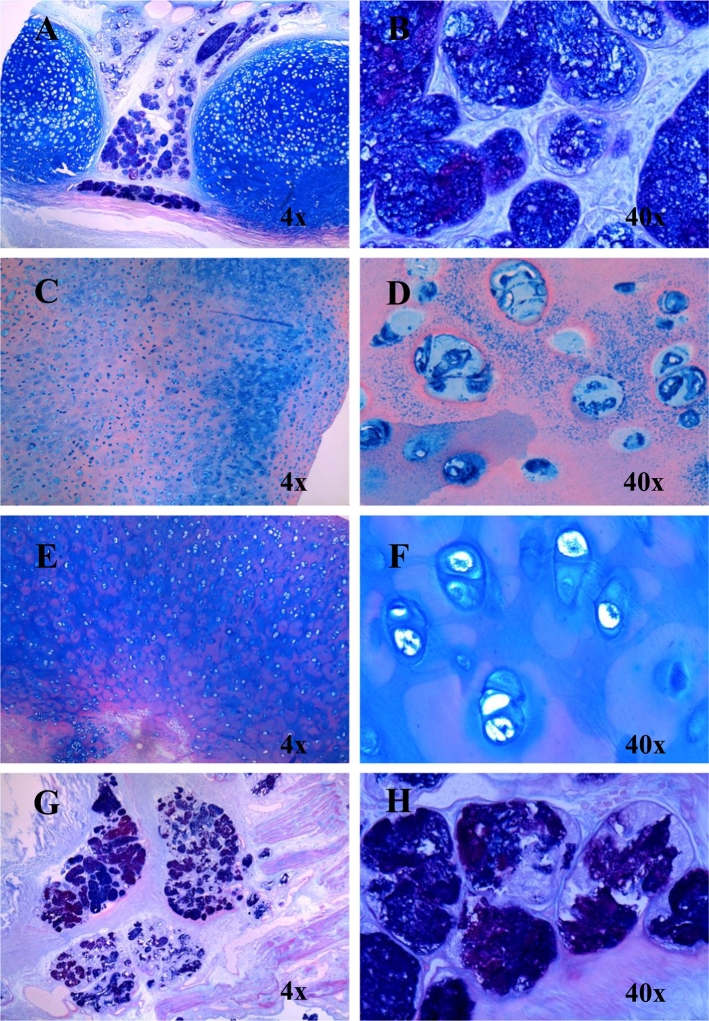
GAGs in the trachea, humerus, knee cartilage, and lung bronchus stained (light microscopy). (A) and (B) Trachea with AB PAS staining shows GAGs colored in dark blue. (C) and (D) The right humerus stained with colloidal iron displays GAGs shown in blue. (E) and (F) Knee cartilage with AB PAS staining shows GAGs colored in blue. (G) and (H) Bronchus in the left lung stained with AB PAS indicates GAGs colored in purple. (For interpretation of the references to colour in this figure legend, the reader is referred to the web version of this article.)

### 3.3 Light microscopy

The majority of chondrocytes in the trachea, humerus, knee cartilage, and lung bronchus were affected by abundant vacuoles (Fig. 6). Trachea was not hypertrophic but had a smooth and elastic surface. The chondrocytes appeared to be enlarged or ballooned. Examination of the affected tissues with AB-PAS and colloidal iron staining indicated that the cartilage was rich with GAGs (Fig. 7).

## 4 Discussion

This autopsied case with the severe phenotype of MPS IVA was assessed clinically, radiographically, and pathologically. We have demonstrated that 1) the patient presented with severe spinal cord compression, and died of tracheal obstruction and successive respiratory failure as a result of the disease, as caused by respiratory muscle weakness from neurological cord deficit due to cord myelopathy, which is commonly attributed to the causes of mortality and morbidity in MPS IVA patients, and 2) the large majority of chondrocytes in the examined locations were enlarged and vacuolated. Our findings suggest that not only is MPS IVA a systemic skeletal disorder, but also involves imbalance of growth with tracheal obstruction, as seen from the patient's large tongue and tortuosity of his trachea, due to the accumulation of GAGs.

The indexed case exhibited worsening respiratory manifestations with time although his cervical decompression and fusion surgery was shown to be successful. The respiratory symptoms, therefore, likely arose from the obstructive disease of his airway, mainly the trachea, as well as from a respiratory muscle weakness arising from cervical spinal cord myelopathy due to the tingling and loss of sensation in his fingers and bilateral lower extremities, and bowel and bladder incontinence.

The current patient exhibited the severe phenotype with smaller weight and stature even compared with averaged MPS IVA males. He stopped growing around the age of 5, which is the most severe end among MPS IVA patients, whose growth typically ceases around the age of 7 or 8 [19]. A study conducted on MPS IVA patients found that the mean height of male patients at 18 years of age was 119.3 ± 22.6 cm (*n* = 116), which corresponded to − 8.0 SD of the height for healthy males [20]. The current patient reached a maximum height of 86.4 cm, correlating to − 13 SD as compared to a healthy male, which is far below the lower end of the mean height for MPS IVA males. This finding indicates that his phenotype was more severe than average male patients with MPS IVA. The study also showed that the mean weight of MPS IVA males at age 18 was 37.6 ± 13.4 kg [19]. The current patient's weight was 19 kg, indicating that he weighed significantly less than average male patients with severe MPS IVA. It was also reported that MPS IVA males had a mean birth length and weight of 52.6 cm and 3.59 ± 0.58 kg, which is slightly larger than the mean birth length and weight of healthy males [19]. Being that the current patient was 43.2 cm long and weighed 2.72 kg at birth and was not born prematurely, this revealed that he was much smaller than the average MPS IVA male patient at birth. The patient's BMI of 25.5 kg/m^2^ is average for MPS IVA males over 18 years of age, who typically have higher BMI's than healthy males [19].

Similar to the autopsied Japanese MPS IVA male patient, the current patient was classified as having the severe phenotype, and both patients were similar in stature, weight, and BMI although the current patient was slightly shorter and weighed less [2]. The Japanese patient presented with smaller stature even when compared among average MPS IVA males, so it is noteworthy that the current patient's stature was 5 cm shorter than the Japanese patient [2]. The Japanese patient received HSCT at the age of 12 years; however, he could not obtain engraftment [2]. Thus, HSCT provides no impact on the autopsy findings, due to the fact that the patient died at 20 years of age. While both patients were found to have severely vacuolated and enlarged chondrocytes, the Japanese patient had aortic valve insufficiency, as well as the appearance of foam cells and macrophages in the lung, aorta, heart valves, heart muscle, trachea, visceral organs, and bone marrow, which were not exhibited in the current patient. It has been found that C6S accumulates in the aorta and heart valves of MPS IVA patients, and that foam cells/macrophages present in the aorta and heart valves contain C6S instead of KS [2], [7]. Thus, it can be inferred that the Japanese patient may have had larger C6S accumulation than the current patient; however, the role of the accumulation of C6S in MPS IVA remains unknown. It should be noted that the Japanese patient died at 20 years of age of acute respiratory distress syndrome and multiple organ failures shortly after receiving occipito-C1-C2 fusion surgery. Difficulty in intubation and extubation was observed, and extubation was impossible after the surgery secondary to severe and ongoing respiratory distress. Retrospectively, the surgical intervention of severe spinal cord compression in the setting of severe tracheal obstruction was an unmet challenge. The current patient's occipito-C1-C2 surgery was performed at the age of 4 years and remained successful; however, the current patient died at 23 years old as a result of respiratory failure with severe tracheal obstruction contributed by respiratory muscle weakness.

In a study examining surgical intervention in MPS IVA patients, four untreated patients at the ages of 20, 23, 28, and 37 years died of respiratory failure with tracheal obstruction [22]. A 20-year old untreated male patient in the same study died from respiratory failure with severe tracheal obstruction after unsuccessful extubation post-cervical fusion surgery. A 16-year old male with severe MPS IVA, who had been receiving ERT for 2.5 years, presented with near-fatal tracheal obstruction, which was alleviated by timely tracheal vascular reconstructive surgery and resulted in great reduction of his respiratory symptoms [3], [11]. Additionally, three patients with severe tracheal obstruction and respiratory distress showed improvement in their symptoms after undergoing successful tracheal reconstructive surgery [22].

These facts indicate that receiving surgery at a young age to alleviate severe orthopedic complications may have advantages over watchful waiting, noting the fact that MPS IVA patients develop difficult airway both due to abnormal upper airway anatomy and later due to tracheal obstruction placing them at high risk for anesthesia [3], [18]. It is especially reasonable to undergo surgical intervention for cervical spinal cord compression earlier to avoid two major surgical interventions at the same time. Since 2015, 10 patients with severe tracheal obstruction (8 out of 10 patients had ERT from 6 months to 5 years) had successful reconstructive surgery of the trachea and showed markedly improved respiratory status and activities of daily living (ADL) post-operation (unpublished). It is critical to monitor the severity of tracheal obstruction once a patient enters their teenage years or if a patient develops new respiratory symptoms.

The current patient demonstrated triangular-shaped tracheal narrowing at the thoracic inlet, with an 81.7% and 66.7% reduction of the minimal cross-sectional area of the trachea from the maximal and median cross-sectional areas, respectively. Taken together with the tortuosity of the trachea, the patient presented with severe tracheal obstruction. This resulted in respiratory failure, which was exacerbated by hypoventilation arising from respiratory muscle weakness from neurological cord deficit due to cord myelopathy.

Undegraded GAGs accumulated in the abundant vacuoles were found in the chondrocytes of the cartilage tissues examined. In addition, the morphology of the chondrocytes was altered, as they appeared ballooned and vacuolated. These findings are consistent with a previous autopsied report of an MPS IVA male, in which vacuolated and irregular chondrocytes were observed [2]. It should be noted that KS, the principal GAG in MPS IVA, is found to accumulate in hyaline cartilage, which makes up the anterior ring of the trachea [3]. Considering that the current patient had enlarged and vacuolated chondrocytes in the trachea, it can be inferred that the accumulation of KS in the trachea may have contributed to the patient's severe tracheal obstruction. Floppiness and a loss of tensile and linear strength of the tracheal wall may result from such deposition of GAGs, which permits focal impression by the brachiocephalic artery, as well as narrowing with neck flexion [3]. The intralysosomal accumulation of GAGs could cause apoptosis of the cells, as well as malfunction of chondrocytes, which will ultimately damage the process of ossification [2], [21]. While there have been indications that clinical and pathological phenotypes appear at an early stage of gestation, this remains unclear, as does the link between the accumulation of GAGs and when such clinical and pathological phenotypes begin.

To understand the pathogenesis of MPS IVA more clearly in several organs, the expression levels of collagens and other molecules must be assessed, as does the relationship between altered molecules and GAG accumulation.

In conclusion, we report here a second autopsied case of an MPS IVA patient with severe tracheal obstruction compounding respiratory distress from muscle weakness originating from cervical cord myelopathy, in which multiple tissues have been analyzed systemically and pathohistologically, and the pathological findings demonstrate a systemic storage disorder with a prominence of vacuolated and enlarged chondrocytes in any location investigated. This is an indication that along with its unique skeletal dysplasia, MPS IVA should be evaluated in a multifaceted approach. In addition, risk factors and appropriate surgical intervention of tracheal obstruction should be investigated to rescue the lives of MPS IVA patients.

## Conflict of interest

All the authors contributed to the article and had no conflict of interest with any other party. Caitlin Doherty, Lauren W. Averill, Mary Theroux, William G. Mackenzie, Christian Pizarro, Robert W. Mason, and Shunji Tomatsu, declare that they have no conflict of interests.

## Contributions to the project

Caitlin Doherty contributed to the planning of the article, a collection of data on the autopsied case, evaluation of pathology, and reporting of the work described.
Lauren W. Averill contributed to the planning of the article, a collection of data on the obstructive airway, evaluation of CT, and reporting of the work described.
Mary Theroux has contributed to the concept of the manuscript, planning of the article, a collection of data on tracheal obstruction, and reporting of the work described.
William G. Mackenzie has contributed to the concept of the manuscript, planning of the article, a collection of data on tracheal obstruction, and reporting of the work described.
Christian Pizarro has contributed to the concept of the manuscript, planning of the article, a collection of data on tracheal obstruction, and reporting of the work described.
Robert W. Mason has contributed to data analysis, and reporting of the work described.
Shunji Tomatsu is a Principal Investigator for this article and has contributed to the concept and planning of the article, a collection of data, and reporting of the work described.
